# Low Incidence of Livestock-Associated Methicillin-Resistant *Staphylococcus aureus* Bacteraemia in The Netherlands in 2009

**DOI:** 10.1371/journal.pone.0073096

**Published:** 2013-08-29

**Authors:** Brigitte A. G. L. van Cleef, Birgit H. B. van Benthem, Anja P. J. Haenen, Thijs Bosch, Jos Monen, Jan A. J. W. Kluytmans

**Affiliations:** 1 Centre for Infectious Disease Control Netherlands, RIVM National Institute for Public Health and the Environment, Bilthoven, The Netherlands; 2 Laboratory for Medical Microbiology and Immunology, St. Elisabeth Hospital, Tilburg, The Netherlands; 3 Laboratory for Medische Microbiology and Infection Control, Amphia Hospital, Breda, The Netherlands; 4 Department of Medical Microbiology and Infection Control, VU University Medical Centre, Amsterdam, The Netherlands; University of Iowa, United States of America

## Abstract

Methicillin-resistant *Staphylococcus aureus* (MRSA) is a worldwide problem in both hospitals and communities all over the world. In 2003, a new MRSA clade emerged with a reservoir in pigs and veal calves: livestock-associated MRSA (LA-MRSA). We wanted to estimate the incidence of bacteraemias due to LA-MRSA using national surveillance data from 2009 in the Netherlands. We found a low incidence of LA-MRSA and MRSA bacteraemia episodes, compared to bacteraemias caused by all *S. aureus* (0.04, 0.18 and 19.3 episodes of bacteraemia per 100,000 inhabitants per year, respectively). LA-MRSA and MRSA were uncommon compared to numbers from other countries as well. MRSA in general and LA-MRSA in specific does not appear to be a public health problem in the Netherlands now. The low incidence of LA-MRSA bacteraemia episodes may best be explained by differences in the populations affected by LA-MRSA versus other MRSA. However, reduced virulence of the strain involved, and the effectiveness of the search and destroy policy might play a role as well.

## Introduction

Methicillin-resistant *Staphylococcus aureus* (MRSA) is a worldwide problem in both hospitals and communities all over the world. *Staphylococcus aureus* (*S. aureus*) is typically a resident of the skin and mucous membranes, but also known for serious infections as wound infections, necrotizing pneumonia, endocarditis, osteomyelitis and sepsis [1].

In 2003, a new MRSA clade with a reservoir in pigs and veal calves emerged: so called livestock-associated MRSA (LA-MRSA) [2]. Until now, high prevalences of LA-MRSA carriage are found in persons in close contact with pigs and veal calves (around 30%) [3,4]. Severe infections have been described occasionally [5] and a few outbreaks have been reported [6–8].

Studies find less virulence genes in LA-MRSA strains [9–12], but the possible effect of this finding has not been studied yet in clinical samples. We wanted to estimate the current incidence of severe infections due to LA-MRSA using a national surveillance database in the Netherlands.

## Methods

This observational study was based on data from the national antibiotic resistance surveillance system (ISIS-AR, www.rivm.nl/cib/themas/isis-ar) which included data from participating microbiological laboratories, and the national MRSA surveillance program (http://mrsa.rivm.nl), where index strains are collected from newly recognized MRSA carriers and/or MRSA infections all over the Netherlands [13].

The 22 participating laboratories in ISIS-AR covered approximately 50% of all hospital beds. Data from 1^st^ January 2009 to 31^st^ December 2009 on *S. aureus* blood isolates with their susceptibility profile were extracted, only the first blood culture isolate per patient per year was included. Since only the material of origin was known, and not the severity of infection, bacteraemias were used as a measure of severe infections. For skin and soft tissue, *S. aureus* can cause a range of infections, from harmless skin lesions to severe deep tissue infections. However, in this database it is impossible to distinguish between these.

ISIS-AR included antibiotic susceptibility profiles, but no information on *spa*-types. The national MRSA surveillance program did have data on *spa*-types and multiple-locus variable number of tandem repeat analysis (MLVA), but did not contain methicillin-sensitive *S. aureus* (MSSA) isolates, and did not specifically contain blood isolates, as every first MRSA positive sample per patient was submitted. Since livestock farmers and other known MRSA risk groups are screened upon admission (for complete national guidelines, see www.wip.nl), it might be possible that a MRSA nasal isolate was submitted to the national MRSA surveillance program, instead of a blood isolate that was found later during hospitalization, resulting in an underestimation for MRSA and LA-MRSA bacteraemias. Therefore, bacteraemia episodes from ISIS-AR were matched to *spa*-types from the national MRSA surveillance database, and missing *spa*-types were retrieved by contacting the individual laboratories. These *spa*-types were used to identify livestock origin, using the criteria defined by Huijsdens et al. [14], and expert opinion from the national reference laboratory. In addition, MLVA was used to determine genetic relatedness between the MRSA strains coming from blood isolates from 2008–2010.

Gender and age from non-LA-MRSA and LA-MRSA bacteraemia episodes were compared with Fisher’s exact test and Wilcoxon-Mann-Whitney test for independent samples with non-normal distributions. For proportions, Wilson confidence intervals (CI) were calculated.

National incidences of all *S. aureus*, MRSA and LA-MRSA in blood cultures were calculated by multiplying the ISIS-AR counts by the proportion of ISIS-admissions to the total number of clinical admissions. The number of clinical admissions of hospitals belonging to the participating laboratories in ISIS-AR was extracted from mandatory annual reports in 2009 (publicly available on www.jaarverslagenzorg.nl), excluding one-day admissions and psychiatric admissions. The total national number of clinical admissions in 2009 was available from Statistics Netherlands (www.cbs.nl), excluding the same two categories. Extrapolation was performed under the assumption that the relation between clinical admissions and population density is the same for laboratories participating in ISIS-AR as those who do not.

The number of *S. aureus* and MRSA carriers in the Netherlands in 2009 were calculated by multiplying the total inhabitant number of the Netherlands (Statistics Netherlands) by 27% [15] or by 0.11% [16], respectively. The number of LA-MRSA carriers in 2009 was calculated by multiplying the total number of persons working in veal calf farming (Statistics Netherlands) by 38% [17], plus the results of multiplying the number of persons working in pig farming (Statistics Netherlands) by 63% (preliminary results from own study group). Chances of a bacteraemia per carrier were calculated by dividing the number of carriers by the number of bacteraemias.

## Results

**Figure 1:**
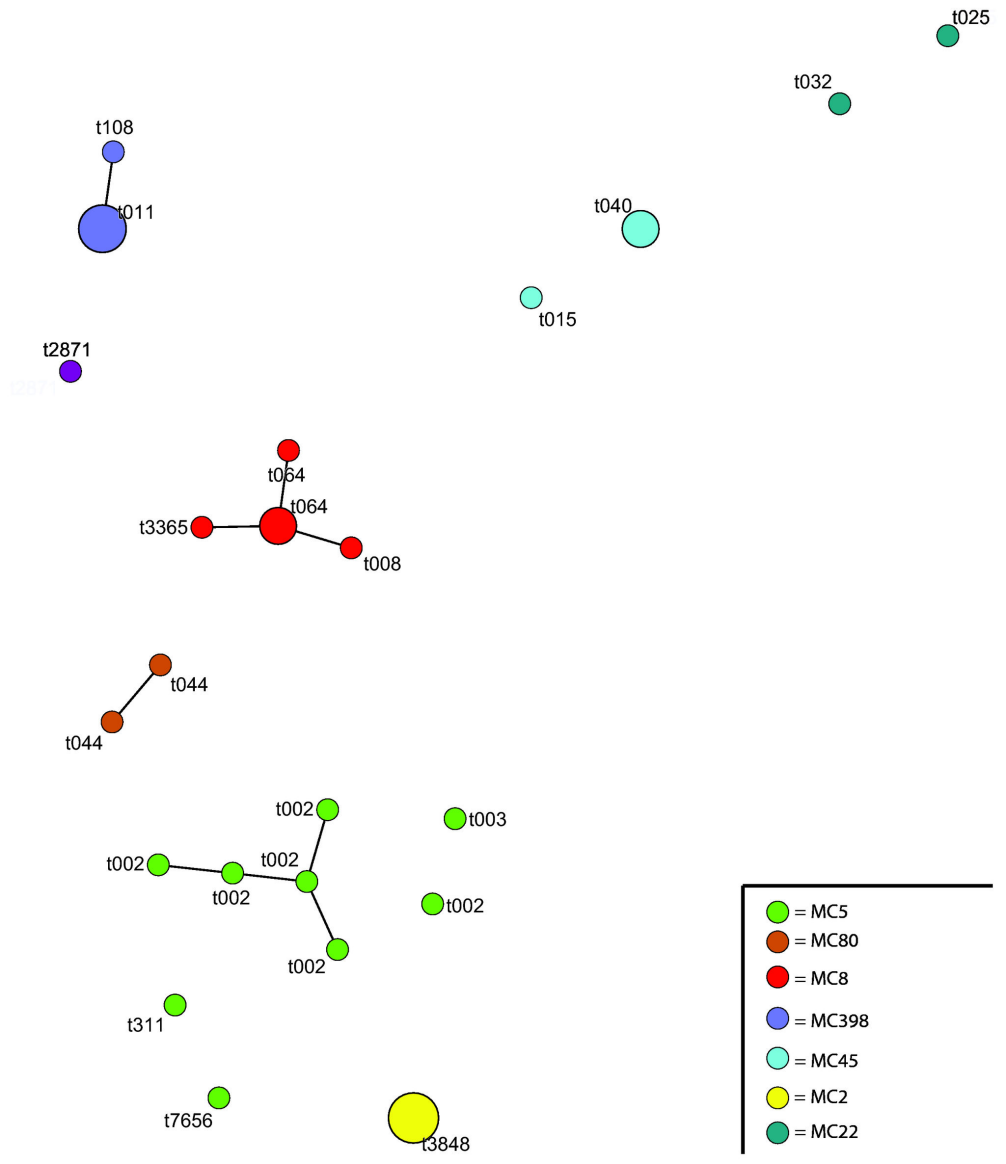
Genetic relatedness of 30 MRSA blood isolates from ISIS-AR from 2008–2010 in the Netherlands. The figure represents as a minimum spanning tree based on MLVA types (MT). Each MT is displayed as a circle with the *spa*-type of the isolate next to it in text, the size denotes the number of isolates, and the color represents the MLVA complex (MC), which are indicated in the legend as well. MC398 stands for MLVA complex 398, which represents the livestock-associated strains.

Data from the year 2009 from the 22 participating labs in ISIS-AR resulted in 1,512 episodes of *S. aureus* bacteraemia. Of the 1,510 episodes with resistance information, 14 were MRSA (14/1,510=0.9%, CI 0.6-1.6%). Of the 13 MRSA bacteraemia episodes with known *spa*-types, three were LA-MRSA (3/13=23%, CI 8-50%, table **1**). MLVA results are shown in Figure **1**. The *spa*-type of isolate 14 could not be traced back in the national MRSA surveillance database or the original laboratory database, and was reported missing. Four isolates (#10-13) showed the same relative rare *spa*-type (t3848), and originated from the same medical centre. These patients are probably part of a hospital outbreak. Gender and age distributions in the LA- and non-LA-MRSA groups were not significantly different (p=1.00 and p=0.46, respectively).

**Table 1:** *Spa*-types of 14 MRSA blood culture isolates from the Netherlands in 2009.

**Isolate no.**	**Gender**	**Age (year)**	***Spa*-type**	**Livestock-associated^a^**
1	Female	74	t002	No
2	Male	79	t002	No
3	Male	70	t011	Yes
4	Female	55	t011	Yes
5	Male	8	t015	No
6	Male	60	t038	No
7	Male	53	t108	Yes
8	Male	0	t740	No
9	Male	76	t3365	No
10	Male	73	t3848	No
11	Male	68	t3848	No
12	Male	69	t3848	No
13	Female	67	t3848	No
14	Male	89	Unknown	Unknown

^a^ Livestock association is determined using Huijsdens et al. [14].

The sum of clinical admissions from hospitals belonging to the participating laboratories in ISIS-AR in 2009 was 903,623, which is 48% of the total of 1,899,000 admissions in the Netherlands. The total inhabitant number for the Netherlands in 2009 was 16,485,787. For *S. aureus*, MRSA and LA-MRSA bacteraemia episodes, incidences are shown in table **2**.

**Table 2:** Incidence of bacteraemia episodes in the Netherlands in 2009.

**Type of *S. aureus***	**Episode counts from ISIS-AR**	**Extrapolation to the Netherlands^a^**	**Incidence per 100,000 inhabitants^b^**
All *S. aureus*	1,512	3,177.5	19.3
MRSA	14	29.4	0.18
LA-MRSA	3	6.3	0.04

^a^ Extrapolation performed by multiplying the ISIS-AR counts by the proportion of ISIS-admissions to the total number of clinical admissions (1,899,000/903,623).

^b^ Incidence per 100,000 inhabitants calculated by dividing the total number for the Netherlands by the total number of inhabitants*100,000 (16,485,787*100,000).

**Figure 2:**
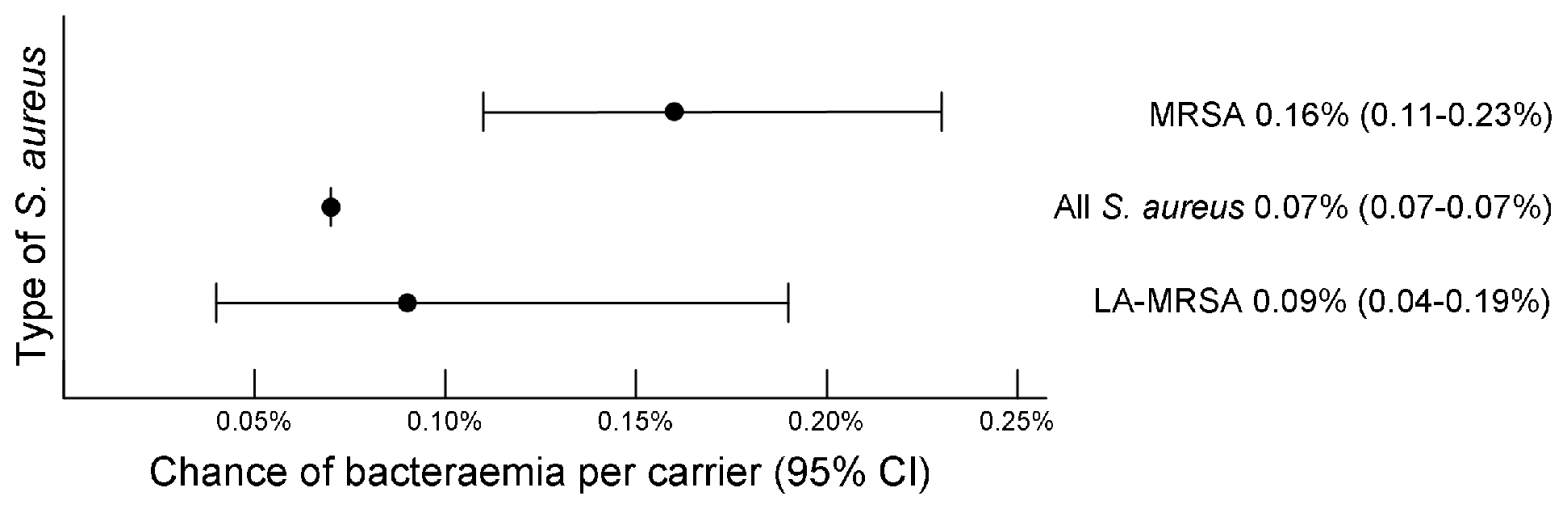
Bacteraemia chances per carrier in the Netherlands in 2009.

The number of *S. aureus* and MRSA carriers in the Netherlands was 4,451,162.5 (16,485,787*0.27), and 18,134.4 (16,485,787*0.0011), respectively. The numbers of persons working in veal calf or pig farming in 2009 were 5620 and 7682.5, respectively, resulting in 6,975.6 LA-MRSA carriers ((5620*0.38) +(7682.5*0.63)). For *S. aureus*, MRSA and LA-MRSA bacteraemia episodes, chances per carrier are shown in Figure **2**.

## Discussion

### Incidences of *S. aureus* bacteraemias

This study based on surveillance data shows that the incidences of LA-MRSA bacteraemias (0.04/100,000 inhabitants) and MRSA bacteraemias (0.18/100,000 inhabitants) are negligible compared to that of *S. aureus* (19.3/100,000 inhabitants). The number of LA-MRSA bacteraemias appears to have been stable over the last years (3 LA-MRSA bacteraemias from 22 laboratories in ISIS-AR in 2009 versus 6 tetracycline/doxycyline-resistant MRSA bacteraemias from 28 laboratories in 2012, data not shown). Methicillin-sensitive *S. aureus* appears to be livestock-associated as well in a substantial number of cases, Verkade and colleagues have recently studied this thus far unknown phenomenon [18].

In a study from Laupland et al., incidences of 2.4 and 26.3 MRSA and *S. aureus* bacteraemias per 100,000 inhabitants were reported for Finland, Australia, Sweden, Canada and Denmark from 2000 to 2008 [19]. Both *S. aureus* and MRSA bacteraemias appear to be less prevalent in the Netherlands, compared to these numbers. Since MRSA-prevalences in the Netherlands are best comparable to those from Northern Europe (Ears-Net, http://ecdc.europa.eu), data from only Finland, Sweden and Denmark were averaged, resulting in 0.4 and 27.1 MRSA and *S. aureus* bacteraemias per 100,000 inhabitants, which is more comparable to our results. Unfortunately, data on LA-MRSA were not available in the study of Laupland et al..

Data from ISIS-AR are very well comparable to data from the national MRSA surveillance program in the Netherlands, where 20 MRSA bacteraemias were counted in 2009, of which 4 were LA-MRSA [20]. Contrary to expectations expressed in the Methods section, there does not seem to be an underestimation of LA-MRSA bacteraemias in this surveillance program.

### Chance of bacteraemia per carrier

The chance for an MRSA carrier to develop a bacteraemia appears to be significantly higher compared to carriers of *S. aureus*, since both confidence intervals do not overlap. For LA-MRSA, due to low numbers confidence intervals are wide, resulting in no significant differences with either other MRSA or *S. aureus* in general. Nevertheless, there appears to be a trend towards a lower chance for a bacteraemia in LA-MRSA carriers, compared to carriers with other MRSA.

### Livestock-associated MRSA

The low incidence of LA-MRSA bacteraemia, as well as the trend for a lower bacteraemia chance per LA-MRSA carrier could be explained in different ways. First, LA-MRSA strains may be less virulent for humans than other MRSA, as less virulence genes have been reported in these strains [9–12,21]. However, experts worry that the rapid evolution of this specific clade may result in gaining new virulence genes in the near future [22].

Second, the Dutch search and destroy strategy includes an active and effective screening regimen that identifies most patients with LA-MRSA at hospital admission (for complete guidelines see www.wip.nl). Decolonization is a part of this strategy, and may prevent the development of bacteraemia [16].

Third and probably most important, differences in patient characteristics may be responsible for the low incidence of LA-MRSA bacteraemias. Persons carrying LA-MRSA are usually working in the livestock industry, and are healthy persons. Persons carrying other (hospital or community associated) MRSA are in general admitted to a hospital, less healthy and probably more likely to develop invasive disease. In addition, an intact skin, which is considered to be the key determinant for protection against *S. aureus* infections, is more likely in livestock farmers than in inpatients, owing to venous lines, operations etc. [1,23].

### Study limitations

This study made use of available data, which bares some limitations: we chose not to include skin and soft tissue infections, and only looked at bacteraemias, since this is the only direct and unambiguous measure for severe infections that can be derived from the available data. We realize that this might imply a possible underestimation of infections. We advise to include other conditions like severe skin and soft tissue infection and pneumonia in future studies.

In addition, selection bias may count for part of the results. Only about 50% of admissions were covered by ISIS-AR, missing some hospitals in larger cities, with the participating laboratories located more often in pig dense areas. If hospitals in large cities would have more non-LA-MRSA bacteraemia episodes, for example because of more foreign patients, patients that travel or complicated patients, the results of this study would underestimate the true MRSA bacteraemia prevalence, and overestimate the LA-MRSA bacteraemias. We consider this effect to be minimal, as other studies do not indicate that there are large differences in MRSA prevalences in hospitals within the Netherlands [24].

Another potential cause of overestimation of the chance of LA-MRSA bacteraemias is the fact that we might have underestimated the number of LA-MRSA carriers, which we calculated from the number of persons working in veal or pig farms only, excluding professional groups as livestock transporters, slaughterhouse workers and veterinarians. In contrast, a possible underestimation of the chance of LA-MRSA bacteraemias might result from a proportion of ‘carriers’ being only contaminated with LA-MRSA, thus not truly colonized. This is supported by a previous study from this group, that reported that MRSA is frequently present after short-term occupational exposure, but in most cases the strain is lost again after 24 hours [25]. Altogether, we believe that possible variations in the chance of a LA-MRSA bacteraemia are of equal size as the confidence interval that is displayed in Figure **2**.

Lastly, international comparison of LA-MRSA bacteraemias is difficult, since the Netherlands have a unique position with both an extremely low MRSA prevalence and a high number of people working in livestock farming. It appears nevertheless that LA-MRSA currently has a low rate of infections in European countries [26].

## Conclusion

This study found a low incidence of LA-MRSA bacteraemia episodes, which may best be explained by differences in the populations affected by LA-MRSA versus other MRSA. However, reduced virulence of the strain involved, and the effectiveness of the search and destroy policy might play a role as well. At present the impact of MRSA in general and LA-MRSA in particular on bacteraemias for the Dutch population appears to be very limited.
